# Facial Expressions and Ability to Recognize Emotions From Eyes or Mouth in Children

**DOI:** 10.5964/ejop.v11i2.890

**Published:** 2015-05-29

**Authors:** Maria Guarnera, Zira Hichy, Maura I. Cascio, Stefano Carrubba

**Affiliations:** a Faculty of Human and Social Sciences, University of Enna “KORE”, Enna, Italy; b Department of Educational Sciences, University of Catania, Catania, Italy; c School of Psychology, Social Work and Human Sciences, University of West London, London, United Kingdom; University of South Wales, Newport, United Kingdom

**Keywords:** facial expressions, emotions, children, recognizing from eyes, recognizing from mouth, labeling task

## Abstract

This research aims to contribute to the literature on the ability to recognize anger, happiness, fear, surprise, sadness, disgust and neutral emotions from facial information. By investigating children’s performance in detecting these emotions from a specific face region, we were interested to know whether children would show differences in recognizing these expressions from the upper or lower face, and if any difference between specific facial regions depended on the emotion in question. For this purpose, a group of 6-7 year-old children was selected. Participants were asked to recognize emotions by using a labeling task with three stimulus types (region of the eyes, of the mouth, and full face). The findings seem to indicate that children correctly recognize basic facial expressions when pictures represent the whole face, except for a neutral expression, which was recognized from the mouth, and sadness, which was recognized from the eyes. Children are also able to identify anger from the eyes as well as from the whole face. With respect to gender differences, there is no female advantage in emotional recognition. The results indicate a significant interaction ‘gender x face region’ only for anger and neutral emotions.

## Introduction

People gather important information by recognizing others’ facial expressions. Correct identification of this visual information, for example, allows individuals to forecast events and respond to them (Isaacowitz et al., 2007). This important capability allows people to infer others’ emotions, which affects their social behavior (e.g., seeing someone who is angry may lead to a ‘fight or flight’ reaction).

Evolutionary psychologists and cognitive neuroscientists have discussed the role of the recognition of emotional expressions in the social environment at length (Biele & Grabowska, 2006; Ebner & Johnson, 2009; Thomas, De Bellis, Graham, & LaBar, 2007). Some have claimed that faces are perceived as a particular category of stimuli by human beings, unlike any other class of objects, and their main importance is that appropriate interpretation is likely to lead to successful social interactions (Keightley, Chiew, Winocur, & Grady, 2007; Nelson, 2001; Tanaka et al., 2012).

From a Darwinian perspective, the ability to recognize emotional facial expressions is innate. Some studies have provided convincing evidence that facial expressions for the basic emotions are universal (Ekman & Friesen, 1971; Ekman et al., 1987; Ekman, Sorenson, & Friesen, 1969; Elfenbein & Ambady, 2002; Izard, 1971).

Reading emotions accurately on the faces of others is the result of complex processes. Important differences in the developmental pattern of the recognition of the different emotions between early childhood and the end of the pre-school period have been well documented. It has been found that facial expressions of happiness, anger, and sadness are generally recognized at an earlier age than those of fear, surprise and disgust (Gagnon, Gosselin, & Maassarani, 2014).

Whereas children are able to produce facial expressions very early in post-natal life (Caron, Caron, & Meyers, 1982; Haviland & Lelwica, 1987; Oster & Ekman, 1978), the ability to recognize emotions from facial expressions increases with age (Fox, 2001; Gross & Ballif, 1991; Herba & Phillips, 2004; Widen & Russell, 2007). Moreover, it appears that children can identify positive facial emotions earlier and more accurately than negative ones (Boyatzis, Chazan, & Ting, 1993; Camras & Allison, 1985; Widen & Russell, 2003), and their accuracy increases between 3 and 7 years of age (Durand, Gallay, Seigneuric, Robichon, & Baudouin, 2007; Markham & Wang, 1996; Vicari, Reilly, Pasqualetti, Vizzotto, & Caltagirone, 2000). Other studies have reported that there are few interesting changes in facial emotion recognition occurring after the age of 7 (Kirouac, Doré, & Gosselin, 1985) or 10 (Tremblay, Kirouac, & Doré, 1987). Others have found that recognition of facial emotions significantly improves between 6 and 15 years of age and adulthood (Herba, Landau, Russell, Ecker, & Phillips, 2006; Herba & Phillips, 2004; Montirosso, Peverelli, Frigerio, Crespi, & Borgatti, 2010; Vicari, Reilly, Pasqualetti, Vizzotto, & Caltagirone, 2000).

Thus, while there is a great deal of research on facial expression recognition in infancy and early childhood, it is uncertain whether this ability continues to develop over this period. In two different research papers, respectively in 2009 and 2010, Gao and Maurer provided more evidence that children show slow development of correct recognition of emotion, and this ability grows with age. The researchers used stimuli sets differentiated by intensity (20 levels for each emotion) and involved children of 5, 7 and 10 years old in the study. While 5-year old children were as capable as adults in recognizing happy emotional expressions, distinguishing between degrees of intensity in the expressions, they confuse anger, fear and sadness. This ability grows with age, and 10-year old children are as capable as adults in assessing these emotions.

This pattern has been partially confirmed by Mancini, Agnoli, Baldaro, Ricci Bitti, and Surcinelli (2013): in their study, children ranging in age from 8 to 11 years old were asked to complete a facial expression recognition task and to rate their emotional reactions in terms of valence and arousal. To complete the emotion recognition task, participants had to select one of six emotion labels (anger, sadness, happiness, fear, disgust, or neutral) that best described the emotional expression they had just seen. In particular, the study demonstrated that happy and angry expressions were the most recognized emotions, followed by expressions of disgust and neutral expressions, while facial expressions of fear and sadness were significantly less recognized compared to all the other emotions (Mancini et al., 2013).

Other factors, such as gender, may affect accuracy in recognition. In particular, previous literature reviews of studies concerning the recognition of facial expressions in children indicated a slightly higher accuracy in females (Brody & Hall, 2010; McClure, 2000; Montirosso et al., 2010), yet this was not confirmed when gender differences were examined separately for each emotion (Gagnon et al., 2014; Rotter & Rotter, 1988).

Numerous studies, albeit with different methods such as discrimination paradigm, matching procedure and free labeling, have investigated the ability to recognize emotions through the stimulus of the whole face. By contrast, only few studies have tested whether recognition accuracy varies as a function of the area of the face shown to participants. These studies, conducted through different tasks, and involving both children with typical and atypical development, have not produced unequivocal results.

Kestenbaum (1992) explored the use of analytic and holistic modes of processing in the recognition of emotional expressions through the five stimulus types as follows: eyes only, mouth only, combination of mouth and eyes, nose missing, full face. All participants (differentiated into three age groups: 5- and 7-year-olds children and adults) were better at recognizing fear, surprise and anger from the eye area than from the mouth area, whereas for happiness the mouth was better for recognizing the emotion.

In a recent study, Gagnon, Gosselin, Hudon-ven der Buhs, Larocque, and Milliard (2010) found that children’s accuracy in recognizing fear and disgust is affected by the array of expressions they are shown. The results suggested that the most common errors made by school-age children consisted in confusing the expressions of anger and disgust, and those for surprise and fear: the likelihood of these two types of error decreases between 5 and 10 years old.

In order to investigate the nature of the facial information allowing children to recognize facial expressions of anger, fear, disgust and surprise, Gagnon et al. (2014) studied whether children correctly recognize the facial expressions of these emotions from action unit combinations (AUCs) located in the upper, middle, or lower face. More specifically, the researchers showed children partial or complete facial expressions and asked them to say whether they corresponded to a given emotion such as anger, fear, surprise, or disgust. As predicted, in agreement with Kestenbaum (1992) they found that children were more accurate at recognizing fear from the AUC located in the upper face than from that located in the lower face and the pattern of results was similar for anger, but this was only the case among girls.

Regarding the differences between autistic and typically developing children in recognizing emotions, many studies showed that individuals with Autism Spectrum Disorder (ASD) performed as well as typically developing adults when they have to attribute mental states to photographs of the eyes (Back, Ropar, & Mitchell, 2007; Speer, Cook, McMahon, & Clark, 2007; van der Geest, Kemner, Camfferman, Verbaten, & van Engeland, 2002). On the contrary, a considerable amount of research suggests that individuals with ASD perform less well than those without ASD on such tasks, because, on the basis of what has been defined as the poor eye gaze hypothesis, they do not attend to a particular facial region such as the eyes. The poor eye gaze hypothesis in ASD has been confirmed by research using different tasks such as the Discrimination Task (Langdell, 1978), the Eyes Task (Baron-Cohen, Jolliffe, Mortimore, & Robertson, 1997; Baron-Cohen, Wheelwright, Hill, Raste, & Plumb, 2001; Baron-Cohen, Wheelwright, & Jolliffe, 1997) and Eyetracking Task (Bal et al., 2010; Norbury et al., 2009). However, the above studies used traditional facial recognition paradigms.

More recently, Song, Kawabe, Hakoda, and Du (2012) found that ASD children extract salient cues from peoples’ eyes when asked to identify both a person and a happy facial expression in an image, and are as accurate as typically developing children. This ability is evaluated by Bubbles, a probing technique which discloses the source of visual information in the observed object (e.g., eyes or mouth), allowing the researcher to explore the strategies used by an individual exposed to a certain stimulus (e.g., an image of a facial expression). By contrast, Tanaka et al. (2012) used Parts-Wholes Expression that evaluates children’s performances by showing a specific facial region (i.e., the eyes or mouth). They found that children with ASD tend to focus more on the mouth and less on the eyes than typically developing children in recognizing the tested expressions of happiness and anger (Tanaka et al., 2012).

The studies outlined above concerned only some of the six basic emotions. Furthermore, it is important to note that children’s performance in recognizing facial expressions varies according to the different methods used, such as discrimination paradigm, matching and labelling procedures. When children interact with other people, they see their faces and have to retrieve the appropriate emotional concept from their long-term memory. The processing of information involved in a labelling task represents a type of processing similar to the process that takes place in everyday life (Russell, 1994).

This research aims to contribute to the relatively scarce literature on the topic by investigating the nature of the facial information that allows children to recognize expressions of anger, happiness, fear, surprise, sadness, disgust and neutral emotion by using the labeling task. Specifically, it explores, for each emotion which facial region (full face, eyes area, mouth area) makes accurate recognition easier. The human face plays a critical role as a perceptual category (Biele & Grabowska, 2006; Ebner & Johnson, 2009; Thomas et al., 2007), and the infant is already capable of distinguishing the drawing of a face with the details of eyes, nose and mouth properly placed from one with various elements placed asymmetrically (Fantz, 1963). It is, therefore, easy to expect that recognition of any emotion should be simpler if one can see the whole face rather than a specific region (eyes or mouth) only. That said, we were primarily interested to know whether children show differences in ability to recognize facial expression of these emotions from a specific region of the face located in the top or bottom face, and if any difference between specific regions of the face (eyes and mouth) depended on the emotion in question.

For this purpose, a group of 6-7 year-old children, an age in which basic emotional recognition has been acquired if not yet stabilized, was selected. Participants were required to recognize emotions (fear, sorrow, anger, surprise, happiness, disgust and a neutral expression) in a labeling task with three stimulus types as follows: region of the eyes, of the mouth, and full face.

## Methods

### Participants

The participants included 50 children (50 percent boys and 50 percent girls) ranging in age from 6 to 7 years old. They were recruited from a Primary School in Sicily (Italy). Children were in their normal school year and had no history of pervasive developmental disorder or other evidence of developmental disability. The examiner worked together with three teachers from the Primary School, and we obtained the parents’ and the headmaster’s approval to administer the instruments. The experimental procedure was explained to the children, and they were free to participate in this research.

### Stimuli

In order to evaluate the children’s performances, children were asked to identify the emotion conveyed in each of the 42 photographs of actors (7 emotions x 3 stimulus types x 2 genders) representing facial expressions. Each image showed one of the six basic emotions (i.e., happiness, sadness, anger, disgust, surprise, fear) or a neutral expression from the *NimStim Face Stimulus Set* developed and validated by Tottenham et al. (2009) and morphed by Gao and Maurer (2009, 2010) to obtain different intensity photographs of facial expressions. The photographs were coloured, with a 560 x 650 pixels resolution. The actors were trained to display specific facial emotions rather than illustrating particular muscle movements, as prescribed in the F.A.C.S. (Ekman & Friesen, 1978). We selected 42 55%-intensity stimuli from the set provided by Gao and Maurer (2009, 2010), and also manipulated 28 pictures so as to directly isolate only those areas considered informative by the literature (i.e., the regions of mouth and eyes). 2-D windows similar to Gosselin and Schyns’ (2001) Bubbles were produced, but with a wider radius according to the photograph. The intention was not to hide the nostrils, which are considered informative (Blais, Roy, Fiset, Arguin, & Gosselin, 2012). The photographs were proportionally divided according to the type of emotion depicted, the actor’s gender, and the facial area isolated, so that whole-face pictures showed a different subject compared to isolated ones. This division allowed the examiner to administer only new stimuli to the same subject, thereby preventing invalidating learning effects.

### Procedure

Participants were tested individually, in a quiet room, arranged for the experimental procedure. The children sat opposite the examiner, in an adequately lighted room. Furthermore, participants were shown one of 42 photographs at a time, and were asked to indicate the right emotion label from a list of drawings showing verbal labels with their respective representations of 2-D emoticons. The researcher noted the subjects’ answers on a sheet. The photographs appeared in random order. The experimenter explained the tasks as follows: “You will gradually see 42 photographs, showing four actors enacting the facial expressions. You will see either the actors’ full faces, just their eyes or their mouth. You should name the correct emotion”.

## Results

For each participant the sum of correct answers, for each emotion and for each three stimulus types (eyes region, mouth region, full face), was calculated (the scores range from 0 to 2). In order to investigate, for each face region, which emotion is more easily recognized, a repeated ANOVA measure with seven levels (the seven emotions) was carried out for each face region. Concerning whole face, the results showed that there are differences in the recognition of emotions [*F*(6, 294) = 13.06, *p* < .001, η^2^ = .21]. Post hoc analysis indicated that the emotions better recognized from the whole face were happiness, anger, and disgust (among which there are no significant differences), followed by fear (which is not different from disgust); while surprise, neutral emotion, and sadness (among which there are no significant differences) are more difficult to recognize (*ps* < .05). Regarding the eyes region, results also showed that, in this case, there are differences in the recognition of emotions [*F*(6, 294) = 24.96, *p* < .001, η^2^ = .34]. Post hoc analysis showed that the emotions better recognized from the eyes region were anger, followed by sadness (which is not different from neutral emotion); the following emotions were the neutral one, happiness and fear (among which was no significant difference), surprise (not different from fear), and finally disgust (*ps* < .05). Finally, also in the case of the mouth region, results showed that there are differences in the recognition of emotions [*F*(6, 294) = 32.51, *p* < .001, η^2^ = .40]. Post hoc analysis showed that the emotions better recognized from the mouth region were the neutral one, followed by anger, disgust, and happiness (among which is no significant difference); the emotions worse recognized from the mouth region were fear, sadness, and surprise (among which was no significant difference, (*ps* < .05).

In order to investigate the children’s performances in detecting emotions from a specific face region, for each emotion a repeated ANOVA measure, with three levels (whole face, eyes region, and mouth region), was carried out. Results showed that the recognition of each emotion was affected by the face region shown [*Fs*(2, 98) > 6.76, *ps* < .01, η^2^s > .12]. With regard to each emotion, post hoc analysis showed that: anger was better recognized when the whole face or eyes area were presented than when the mouth area was presented. Disgust was better recognized when the whole face was presented than when the mouth area was presented, while it appeared more difficult to recognize disgust presenting the eyes region. Fear, happiness, and surprise were better recognized when the whole face was presented compared to the eyes or mouth regions. Sadness was better recognized when the eyes region was presented, followed by presentation of the whole face, while the recognition of sadness appeared more difficult when the mouth area was presented. Finally, neutral emotion was better recognized when the mouth area was presented, compared to the whole face or eyes area (Table 1).

**Table 1:** Children’s Performances in Detecting Emotions From a Specific Face Region.

Emotion	Whole face		Eyes area		Mouth area	
	*M*	*SD*	*M*	*SD*	*M*	*SD*
Anger	1.60_a_	0.61	1.60_a_	0.61	1.20_b_	0.64
Disgust	1.52_a_	0.58	0.24_b_	0.48	1.18_c_	0.48
Fear	1.34_a_	0.69	0.80_b_	0.67	0.70_b_	0.74
Happiness	1.62_a_	0.57	0.90_b_	0.58	1.04_b_	0.53
Sadness	0.90_a_	0.58	1.12_b_	0.63	0.50_c_	0.54
Surprise	1.04_a_	0.75	0.60_b_	0.76	0.44_b_	0.61
Neutral	0.92_a_	0.75	0.92_a_	0.80	1.74_b_	0.49

*Note*. For each row, means with differing subscripts are significantly different, *p* < .05.

In order to test the combined effect of gender and face region, a 2 × 3 ANOVA, with gender as between-subjects factors, and face region as a within-subjects factor, was carried out. Results indicated a significant interaction “gender x face region” only for anger [*F*(2, 96) = 4.65, *p* < .02, η^2^ = .08] and neutral emotion [*F*(2, 96) = 4.28, *p* < .02, η^2^ = .08]. With regard to anger, post hoc analysis showed that male participants recognized angry from the eyes area and the mouth area (eyes: *M* = 1.80, *SD* = 0.41; mouth: *M* = 1.40, *SD* = 0.58) better than female participants (eyes: *M* = 1.40, *SD* = 0.71; mouth: *M* = 1.00, *SD* = 0.64; *ps* < .05). Regarding neutral emotion, results of post hoc analysis showed that male participants recognized this emotion from the whole face (*M* = 1.28, *SD* = 0.79) better than female participants (*M* = 0.56, *SD* = 0.51, *p* < .001). Moreover, male participants recognise anger better when the eyes area, rather than the face or mouth area is presented, while females recognise this emotion better from the mouth region than from the whole face (*ps* < .05). In the case of neutral emotion, both genders recognise it better from the mouth area than from the whole face or eyes area (*ps* < .05).

## Discussion and Conclusions

The aim of this survey is to investigate the nature of the facial information which allows 6-7 year-old children to recognize expressions of anger, happiness, fear, surprise, sadness, disgust and neutral emotion by using a labelling task. Photographs representing these emotions through three stimulus types, the whole face, the eyes only area or the mouth only area were presented.

A preliminary analysis was addressed to investigate the differences between the emotions for each stimulus type (whole face, eyes region, mouth region).

The emotions better recognized from the whole face were happiness, anger, and disgust, followed by fear; while surprise, neutral emotion, and sadness were more difficult to recognize. Gao and Maurer (2009, 2010) and Mancini et al. (2013) demonstrated that children are capable of recognizing emotional expressions, but they confuse fear and sadness. This ability increases with age, so it is expected that this group of 6-7 year olds will become more expert with age. These results can be linked to evolutionary significance: decoding is especially important in children as they need safety and protection, so they easily detect emotions indispensable in terms of survival (such as happiness, anger, disgust, and fear) while they find more difficult to recognize surprise. This result can be linked to cognitive development too: facial emotion recognition gradually improves throughout normal development, so a poor accuracy in recognizing facial expressions such as surprise, sadness, and neutral could be related to the age of the group. In this context, Mancini et al. (2013) demonstrated that the recognition of neutral expressions, defined as ‘emotionally ambiguous expressions’, is characterized by an evident increase in accuracy from 8 to 11 years of age.

It is interesting to note that there are differences between the emotions with respect both to the region of the eyes and that of the mouth.

According to our results, it seems that, for both regions of the face, anger is one of the easiest emotions to identify, while surprise is among the most difficult to recognize. Evolutionally speaking, anger may be defined as the most crucial emotion when it comes to recognize others’ intentions, in order to avoid threats, and this circumstance could account for its being the easiest to detect than any other.

Alternatively, as far as surprise is concerned, as Maassarani, Gosselin, Montembeault, and Gagnon (2014) suggest, the difficulty of its recognition could depend on the fact that some emotions may follow one another in a short period. Many situations that bring happiness may result from an unexpected source, and so happiness may be experienced immediately after a surprise. The same applies to the relation between fear and surprise. The temporal proximity between surprise and these two emotions, therefore, could make recognition of surprise harder for children.

Overall, these differences between the emotions on the one hand support Ekman’s idea that each emotion is configured for a specific muscle pattern, and on the other suggest that both the eyes and the mouth are relevant in facial emotions, each in a different way. Moreover, the fact that the role of a specific region is different depending on the emotions is in line with a study by Song et al. (2012), in which he argues that it is easier for children with ASD to gaze at another’s eyes when processing positive emotions than in the case when the emotions are negative.

Subsequent analysis has allowed us to compare differences between the different stimuli (full face, eyes region, mouth region) for each emotion.

First of all, considering the critical role that the human face assumes as a perceptual category (Biele & Grabowska, 2006; Ebner & Johnson, 2009; Thomas et al., 2007), it was fairly predictable that children’s ability to recognize emotions should be better when they see the whole face rather than a specific region (eyes or mouth). However, our concern was to investigate whether for the children there would be differences in recognizing the emotions from a specific facial region located in the upper or lower face, and if any difference between specific facial regions (eyes or mouth) may depend on the emotion in question.

The findings of this survey seem to indicate that children generally recognize the basic facial expressions better when pictures represent the whole face, except for a neutral expression, which is recognized from the mouth, and sadness from the eyes. Moreover, children are able to identify anger from the eyes as well as from the whole face. It is quite counter-intuitive that the whole face was not always best for children in identifying emotions, as one might think that ‘the more information the better’ would apply regardless of emotion. This would suggest that directing the focus to such isolated cues may have facilitated detection due to the absence of potentially confounding cues.

Our results, in agreement with those reported by Gagnon et al. (2014) indicate that children appreciate emotions through a specific facial area and, more specifically, from the upper face.

With respect to the areas of the face (upper or lower) that best convey the basic emotions, this study indicates that 6-7 year-old children’s performances change depending on the emotion. In agreement with the results reported by Kestenbaum (1992) and Gagnon et al. (2014), but in contrast with those of Boucher and Ekman (1975) related to college students, which showed no differences, these results seem to indicate that children are more accurate at recognizing anger from the regions located in the upper face than from the lower face. According to Boucher and Ekman (1975), sadness and disgust are better recognized, respectively, from the upper area than from the lower and from the lower area than from the upper. In three instances, happiness, surprise and fear, recognition from the upper face was as good as recognition from the lower. As for happiness, this result is in agreement with Boucher and Ekman (1975) and with Song et al. (2012). As for surprise, similar results were shown by Boucher and Ekman (1975) and partly also by Kestenbaum (1992) with regard to children of 5 years old. They are, however, in contrast with the results reported by Gagnon et al. (2014) and with those of Kestenbaum (1992) with regard to older children, which showed better performances in recognizing from the eyes area than from that of the mouth. As for fear, Gagnon et al. (2014) found similar results in children of 5 years old. Boucher and Ekman (1975), Kestenbaum (1992), Gagnon et al. (2014), meanwhile, with regard to older children, found better performances in recognizing it from the area of the eyes than the mouth.

Apart from comparison with other studies, which could be affected by methodological differences, the results indicate some differences between the regions of the eyes and mouth in facilitating the recognition of emotions. It could be that the facial regions have different roles according to the sensory-motor correlates (voluntary and involuntary) that characterize the various emotions. For example, the region of the eyes is more relevant in the case of anger because it is associated with movements related to frowning, with arching the eyebrows, or visual sensations like ’bloodshot eyes’, ‘a red mist descends’, or ‘to be blinded by rage’. It is worth noting, in any case that, compared to the other emotions, anger is among the easiest to recognize from the mouth. Anger, in fact, is also associated with mouth movements such as tooth grinding, pouting, tightening the jaws or opening the mouth to bite or scream.

The eyes region is very relevant in the case of sadness because this emotion is often associated with the tactile sensation of tears and the ocular reflexes that produce them. By contrast, the region of the mouth is central in the case of disgust because it is associated with the sensation of taste in the mouth and the movements, which can be unintentional, performed to eliminate unpalatable food.

Interestingly, except for happiness, children recognize emotions for which no differences appear between the areas of the eyes and that of the mouth (surprise and fear), at a later developmental stage (Gagnon et al., 2014). It would be interesting to verify, using the same method, if adults too show the same pattern or if a region becomes more important than another.

Finally, children show better performance in recognizing a neutral expression from the mouth area than from that of the eyes (and full face). This last finding cannot be compared with previous results, because the researchers did not investigate children’s ability to recognize neutral emotions. Further investigation could clarify the overall results of our study with regard to recognition of this facial expression. On the one hand, in fact, as affirmed above, this is among the most difficult to recognize of the entire face. In this regard, other studies have described neutral faces as emotionally ambiguous expressions and children aged 4-8 have been defined as characterized by a low accuracy of recognition for this expression (Waters, Neumann, Henry, Craske, & Ornitz, 2008). Mancini et al. (2013) argued that the recognition of neutral expressions is characterized by an evident increase in accuracy from 8 to 11 years of age. On the other hand, neutral expression is one of the most easily recognized from the region of the eyes, and the easiest of all emotions to be recognized from the mouth. Subsequent analysis, based on the quality of the wrong answers, would allow us to test the hypothesis that, while when the children see the whole face, they find indicators of some emotion in the complex of information; when they see only a part of the face - this typically being the most difficult task - they more often tend to say neutral. If this is the case, it would mean that the correct answer does not correspond to real recognition of the neutral expression, but to the major frequency of a neutral answer in the case of partial stimuli.

With respect to gender differences, according to Mancini et al. (2013) and Gagnon et al. (2014), there is no female advantage in emotional recognition: this survey’s results indicate a significant interaction ‘gender x face region’ (full face, eyes only, mouth only) only for anger and neutral emotion. More specifically, male participants recognize anger from the eyes better than females, who recognize this emotion from the mouth. In addition, both genders recognise a neutral emotion better from the mouth than from the whole face or eyes. Further research could try to replicate such results in order to interpret potential differences of gender like those highlighted by us. For instance, taking into account the actual emotions attributed by these participants during the test could help in interpreting such findings in terms of gender differences.

On the basis of the outcome of our study and those of previous research, some considerations can be advanced.

Differences emerged between the emotions relating to each stimulus, on the one hand support the idea of Ekman that each emotion is configured for a specific muscle pattern, and on the other suggest that both the eyes and the mouth areas are relevant in a different manner for each emotion.

It is important to underline that we compared six emotions and a neutral expression with the aim of investigating if children show differences in recognizing emotions from a specific area of the face (eyes and mouth).

Overall, all studies, including this one, demonstrate the ability of children to recognize emotions from partial regions of the face, particularly from that of the eyes. However, among the different studies, for each emotion, the results are not always consistent with reference to the relevance of one region compared to another (for example, regarding surprise, some studies indicate the prevalence of the mouth area, others of the eyes, or even no difference).

However, despite of results partially different, perhaps attributable to the different research methods used, the capacity displayed by the children to recognize emotions even from partial areas of the face and, consequently, the importance of the specific regions in conveying information for the recognition of emotions give rise to the question about the underlying processes. As suggested by Gagnon et al. (2014), the partial regions of the face may only be signals, in themselves meaningless, useful for the recovery from long-term memory of the entire face; or, according to the componential model proposed by Scherer (2001), have their own meaning, useful in the appraisal process involved in recognizing emotion. In this case the different results of the studies may reflect real individual differences based on different experiences linked to the formation of the concept of each emotion. According to Widen and Russell (2013), the concept of an emotion corresponds to the relation between several components such as children’s knowledge of the causes, of its behavioral consequences, of its facial, vocal, and behavioral correlates, of its bodily changes, and of the words used to name it. It could therefore be argued that, according to the different experiences, it is possible to add different facial correlates to the concept of a single emotion. For instance, as far as surprise is concerned, both the raising of the upper eyelids and the opening of the mouth are motor components associated with appraisals of novelty. Which of these components is associated with the concept of surprise is thought to depend upon the regularity with which they occur to the children during the process of concept formation. Further studies aimed at resolving these issues would be interesting.

Moreover, subsequent studies characterized by comparison between adults and children in the area of the decoding of the emotional expressions of faces from partial information (eyes and mouth) could verify if the pattern in the difference between the specific regions remains unchanged in adults too.

In addition, future studies, using the same method, with autistic or alexithymic subjects, could also be useful in the clinical setting, since the results could be used to prepare individualized training for the recognition of emotions.

## References

[r1] Back E. Ropar D. Mitchell P. (2007). Do the eyes have it? Inferring mental states from animated faces in autism. Child Development, 78(2), 397–411. 10.1111/j.1467-8624.2007.01005.x 17381780

[r2] Bal E. Harden E. Lamb D. Van Hecke A. V. Denver J. W. Porges S. W. (2010). Emotion recognition in children with autism spectrum disorders: Relations to eye gaze and autonomic state. Journal of Autism and Developmental Disorders, 40(3), 358–370. 10.1007/s10803-009-0884-3 19885725

[r3] Baron-Cohen S. Jolliffe T. Mortimore C. Robertson M. (1997). Another advanced test of theory of mind: Evidence from very high functioning adults with autism or Asperger syndrome. Journal of Child Psychology and Psychiatry, 38(7), 813–822. 10.1111/j.1469-7610.1997.tb01599.x 9363580

[r4] Baron-Cohen S. Wheelwright S. Hill J. Raste Y. Plumb I. (2001). The “Reading the Mind in the Eyes” Test revised version: A study with normal adults, and adults with Asperger syndrome or high-functioning autism. Journal of Child Psychology and Psychiatry, 42(2), 241–251. 10.1111/1469-7610.00715 11280420

[r5] Baron-Cohen S. Wheelwright S. Jolliffe T. (1997). Is there a “language of the eyes”? Evidence from normal adults, and adults with autism or Asperger syndrome. Visual Cognition, 4(3), 311–331. 10.1080/713756761

[r6] Biele C. Grabowska A. (2006). Sex differences in perception of emotion intensity in dynamic and static facial expressions. Experimental Brain Research, 171(1), 1–6. 10.1007/s00221-005-0254-0 16628369

[r7] Blais C. Roy C. Fiset D. Arguin M. Gosselin F. (2012). The eyes are not the window to basic emotions. Neuropsychologia, 50(12), 2830–2838. 10.1016/j.neuropsychologia.2012.08.010 22974675

[r8] Boucher J. D. Ekman P. (1975). Facial areas and emotional information. Journal of Communication, 25(2), 21–29. 10.1111/j.1460-2466.1975.tb00577.x 1127138

[r9] Boyatzis C. J. Chazan E. Ting C. Z. (1993). Preschool children’s decoding of facial emotions. The Journal of Genetic Psychology, 154(3), 375–382. 10.1080/00221325.1993.10532190 8245911

[r10] Brody, L. R., & Hall, J. A. (2010). Gender and emotion in context. In M. Lewis, J. M. Haviland-Jones, & L. Feldman Barrett (Eds.), *Handbook of emotions* (3rd ed., pp. 395-408). New York, NY: The Guilford Press.

[r11] Camras L. A. Allison K. (1985). Children’s understanding of emotional facial expressions and verbal labels. Journal of Nonverbal Behavior, 9(2), 84–94. 10.1007/BF00987140

[r12] Caron R. F. Caron A. J. Meyers R. S. (1982). Abstraction of invariant face expressions in infancy. Child Development, 53, 1008–1015. 10.2307/1129141 7128251

[r13] Durand K. Gallay M. Seigneuric A. Robichon F. Baudouin J.-Y. (2007). The development of facial emotion recognition: The role of configural information. Journal of Experimental Child Psychology, 97(1), 14–27. 10.1016/j.jecp.2006.12.001 17291524

[r14] Ebner N. C. Johnson M. K. (2009). Young and older emotional faces: Are there age group differences in expression identification and memory? Emotion, 9(3), 329–339. 10.1037/a0015179 19485610 PMC2859895

[r15] Ekman P. Friesen W. V. (1971). Constants across cultures in the face and emotion. Journal of Personality and Social Psychology, 17(2), 124–129. 10.1037/h0030377 5542557

[r16] Ekman, P., & Friesen, W. V. (1978). *Facial action coding system: A technique for the measurement of facial movement*. Palo Alto, CA: Consulting Psychologists Press.

[r17] Ekman P. Friesen W. V. O’Sullivan M. Chan A. Diacoyanni-Tarlatzis I. Heider K. Tzavaras A. (1987). Universals and cultural differences in the judgments of facial expressions of emotion. Journal of Personality and Social Psychology, 53(4), 712–717. 10.1037/0022-3514.53.4.712 3681648

[r18] Ekman P. Sorenson E. R. Friesen W. V. (1969). Pan-cultural elements in the facial displays of emotion. Science, 164(3875), 86–88. 10.1126/science.164.3875.86 5773719

[r19] Elfenbein H. A. Ambady N. (2002). On the universality and cultural specificity of emotion recognition: A meta-analysis. Psychological Bulletin, 128(2), 203–235. 10.1037/0033-2909.128.2.203 11931516

[r20] Fantz R. L. (1963). Pattern vision in newborn infants. Science, 140(3564), 296–297. 10.1126/science.140.3564.296 17788054

[r21] Fox, J. (2001). *Identifying emotions in faces: A developmental study* Washington, DC: Intel Science Talent Search. Retrieved from http://psych.nyu.edu/pelli/docs/JeremyFoxIntel.pdf

[r22] Gagnon M. Gosselin P. Hudon-ven der Buhs I. Larocque K. Milliard K. (2010). Children’s recognition and discrimination of fear and disgust facial expressions. Journal of Nonverbal Behavior, 34(1), 27–42. 10.1007/s10919-009-0076-z

[r23] Gagnon M. Gosselin P. Maassarani R. (2014). Children's ability to recognize emotions from partial and complete facial expressions. The Journal of Genetic Psychology, 175(5), 416–430. 10.1080/00221325.2014.941322 25271818

[r24] Gao X. Maurer D. (2009). Influence of intensity on children’s sensitivity to happy, sad, and fearful facial expressions. Journal of Experimental Child Psychology, 102(4), 503–521. 10.1016/j.jecp.2008.11.002 19124135

[r25] Gao X. Maurer D. (2010). A happy story: Developmental changes in children’s sensitivity to facial expressions of varying intensities. Journal of Experimental Child Psychology, 107(2), 67–86. 10.1016/j.jecp.2010.05.003 20542282

[r26] Gosselin F. Schyns P. G. (2001). Bubbles: A technique to reveal the use of information in recognition tasks. Vision Research, 41(17), 2261–2271. 10.1016/S0042-6989(01)00097-9 11448718

[r27] Gross A. L. Ballif B. (1991). Children’s understanding of emotion from facial expressions and situations: A review. Developmental Review, 11(4), 368–398. 10.1016/0273-2297(91)90019-K

[r28] Haviland J. M. Lelwica M. (1987). The induced affect response: 10-week-old infants’ responses to three emotion expressions. Developmental Psychology, 23(1), 97–104. 10.1037/0012-1649.23.1.97

[r29] Herba C. M. Landau S. Russell T. Ecker C. Phillips M. L. (2006). The development of emotion-processing in children: Effects of age, emotion, and intensity. Journal of Child Psychology and Psychiatry, 47(11), 1098–1106. 10.1111/j.1469-7610.2006.01652.x 17076748

[r30] Herba C. Phillips M. (2004). Annotation: Development of facial expression recognition from childhood to adolescence: Behavioural and neurological perspectives. Journal of Child Psychology and Psychiatry, 45(7), 1185–1198. 10.1111/j.1469-7610.2004.00316.x 15335339

[r31] Isaacowitz D. M. Löckenhoff C. E. Lane R. D. Wright R. Sechrest L. Riedel R. Costa P. T. (2007). Age differences in recognition of emotion in lexical stimuli and facial expressions. Psychology and Aging, 22(1), 147–159. 10.1037/0882-7974.22.1.147 17385991

[r32] Izard, C. E. (1971). *The face of emotion*. New York, NY: Appleton-Century-Crofts.

[r33] Keightley M. L. Chiew K. S. Winocur G. Grady C. L. (2007). Age-related differences in brain activity underlying identification of emotional expressions in faces. Social Cognitive and Affective Neuroscience, 2(4), 292–302. 10.1093/scan/nsm024 18985135 PMC2566756

[r34] Kestenbaum R. (1992). Feeling happy versus feeling good: The processing of discrete and global categories of emotional expressions by children and adults. Developmental Psychology, 28(6), 1132–1142. 10.1037/0012-1649.28.6.1132

[r35] Kirouac, G., Doré, F. Y., & Gosselin, F. (1985). The recognition of facial expressions of emotions. In R. E. Tremblay, M. A. Provost, & F. F. Strayer (Eds.), *Éthologie et développement de l’enfant* (pp. 131-147). Paris, France: Stock.

[r36] Langdell T. (1978). Recognition of faces: An approach to the study of autism. Journal of Child Psychology and Psychiatry, 19(3), 255–268. 10.1111/j.1469-7610.1978.tb00468.x 681468

[r37] Maassarani R. Gosselin P. Montembeault P. Gagnon M. (2014). French-speaking children’s freely produced labels for facial expressions. Frontiers in Psychology, 5, . 10.3389/fpsyg.2014.00555 24926281 PMC4044975

[r38] Mancini G. Agnoli S. Baldaro B. Ricci Bitti P. E. Surcinelli P. (2013). Facial expressions of emotions: Recognition accuracy and affective reactions during late childhood. The Journal of Psychology, 147(6), 599–617. 10.1080/00223980.2012.727891 24199514

[r39] Markham R. Wang L. (1996). Recognition of emotion by Chinese and Australian children. Journal of Cross-Cultural Psychology, 27(5), 616–643. 10.1177/0022022196275008

[r40] McClure E. B. (2000). A meta-analytic review of sex differences in facial expression processing and their development in infants, children, and adolescents. Psychological Bulletin, 126(3), 424–453. 10.1037/0033-2909.126.3.424 10825784

[r41] Montirosso R. Peverelli M. Frigerio E. Crespi M. Borgatti R. (2010). The development of dynamic facial expression recognition at different intensities in 4- to 18-year-olds. Social Development, 19(1), 71–92. 10.1111/j.1467-9507.2008.00527.x

[r42] Nelson C. A. (2001). The development and neural bases of face recognition. Infant and Child Development, 10(1-2), 3–18. 10.1002/icd.239

[r43] Norbury C. F. Brock J. Cragg L. Einav S. Griffiths H. Nation K. (2009). Eye-movement patterns are associated with communicative competence in autistic spectrum disorders. Journal of Child Psychology and Psychiatry, 50(7), 834–842. 10.1111/j.1469-7610.2009.02073.x 19298477

[r44] Oster, H., & Ekman, P. (1978). Facial behavior in child development. In W. A. Collins (Ed.), *Minnesota Symposia on Child Psychology* (Vol. 11, pp. 231-276). Hillsdale, NJ: Erlbaum.

[r45] Rotter N. G. Rotter G. S. (1988). Sex differences in the encoding and decoding of negative facial emotions. Journal of Nonverbal Behavior, 12(2), 139–148. 10.1007/BF00986931

[r46] Russell J. A. (1994). Is there universal recognition of emotion from facial expressions? A review of the cross-cultural studies. Psychological Bulletin, 115(1), 102–141. 10.1037/0033-2909.115.1.102 8202574

[r47] Scherer, K. R. (2001). Appraisal considered as a process of multilevel sequential checking. In K. R. Scherer, A. Schorr, & T. Johnstone (Eds.), *Appraisal processes in emotion: Theory, methods, research* (pp. 92-120). New York, NY: Oxford University Press.

[r48] Song Y. Kawabe T. Hakoda Y. Du X. (2012). Do the eyes have it? Extraction of identity and positive expression from another’s eyes in autism, probed using “Bubbles”. Brain & Development, 34(7), 584–590. 10.1016/j.braindev.2011.09.009 21999967

[r49] Speer L. L. Cook A. E. McMahon W. M. Clark E. (2007). Face processing in children with autism: Effects of stimulus contents and type. Autism, 11(3), 265–277. 10.1177/1362361307076925 17478579

[r50] Tanaka J. W. Wolf J. M. Klaiman C. Koenig K. Cockburn J. Herlihy L. Schultz R. T. (2012). The perception and identification of facial emotions in individuals with autism spectrum disorders using the Let’s Face It! Emotion Skills Battery. The Journal of Child Psychology and Psychiatry, and Allied Disciplines, 53(12), 1259–1267. 10.1111/j.1469-7610.2012.02571.x 22780332 PMC3505257

[r51] Thomas L. A. De Bellis M. D. Graham R. LaBar K. S. (2007). Development of emotional facial recognition in late childhood and adolescence. Developmental Science, 10(5), 547–558. 10.1111/j.1467-7687.2007.00614.x 17683341

[r52] Tottenham N. Tanaka J. W. Leon A. C. McCarry T. Nurse M. Hare T. A. Nelson C. (2009). The NimStim set of facial expressions: Judgments from untrained research participants. Psychiatry Research, 168(3), 242–249. 10.1016/j.psychres.2008.05.006 19564050 PMC3474329

[r53] Tremblay C. Kirouac G. Doré F. Y. (1987). The recognition of adults’ and children’s facial expressions of emotions. The Journal of Psychology, 121(4), 341–350. 10.1080/00223980.1987.9712674

[r54] van der Geest J. N. Kemner C. Camfferman G. Verbaten M. N. van Engeland H. (2002). Looking at images with human figures: Comparison between autistic and normal children. Journal of Autism and Developmental Disorders, 32(2), 69–75. 10.1023/A:1014832420206 12058845

[r55] Vicari S. Reilly J. S. Pasqualetti P. Vizzotto A. Caltagirone C. (2000). Recognition of facial expressions of emotions in school-age children: The intersection of perceptual and semantic categories. Acta Paediatrica, 89(7), 836–845. 10.1111/j.1651-2227.2000.tb00392.x 10943968

[r56] Waters A. M. Neumann D. L. Henry J. Craske M. G. Ornitz E. M. (2008). Baseline and affective startle modulation by angry and neutral faces in 4–8-year-old anxious and non-anxious children. Biological Psychology, 78(1), 10–19. 10.1016/j.biopsycho.2007.12.005 18243481

[r57] Widen S. C. Russell J. A. (2003). A closer look at preschoolers' freely produced labels for facial expressions. Developmental Psychology, 39(1), 114–128. 10.1037/0012-1649.39.1.114 12518813

[r58] Widen, S. C., & Russell, J. A. (2007). *Children’s understanding of scripts for basic-level vs. social emotions*. Poster presented at the 2007 Biennial Meeting of the Society for Research in Child Development, Boston, MA.

[r59] Widen S. C. Russell J. A. (2013). Children’s recognition of disgust in others. Psychological Bulletin, 139(2), 271–299. 10.1037/a0031640 23458434

